# CLeaFA: Development and Validation of the Clinical Leadership Contextual Factors Self‐Assessment Tool in Belgian Hospitals

**DOI:** 10.1155/jonm/9019085

**Published:** 2026-09-30

**Authors:** Sabrina Nachtergaele, Lukas Billiau, Nele De Roo, Kristof Eeckloo, Annelies Bockstael, Mieke Embo, Veerle Duprez

**Affiliations:** ^1^ Research and Innovation Centre Health and Care Artevelde University of Applied Sciences Ghent Belgium; ^2^ Strategic Policy Unit Ghent University Hospital Ghent Belgium uzgent.be; ^3^ Department of Public Health and Primary Care Faculty of Medicine and Health Sciences Ghent University Ghent Belgium ugent.be; ^4^ Department of Education and Department of Work Organisation and Society Faculty of Psychology and Educational Sciences Ghent University Ghent Belgium ugent.be; ^5^ Department of Nursing Ghent University Hospital Ghent Belgium uzgent.be

**Keywords:** clinical leadership, contextual factors, leadership development, organizational culture, psychometrics, self-assessment, validation study

## Abstract

**Background:**

Healthcare professionals who actively engage in clinical leadership contribute to a culture of continuous improvement of quality of care. However, the enactment of clinical leadership strongly depends on contextual factors. Although previous research has identified several enabling contextual factors, there is no validated self‐assessment tool to systematically capture and analyze these factors in practice. This gap limits the ability to assess and support clinical leadership development.

**Objective:**

To develop and validate a self‐assessment tool that identifies contextual factors influencing the development of clinical leadership among healthcare professionals.

**Methods:**

The Clinical Leadership Contextual Factors (CLeaFA) tool was developed and validated through a three‐phase process: (1) item development informed by qualitative focus group data, (2) content validation via a Delphi procedure with experts, and (3) psychometric validation using exploratory factor analysis, confirmatory factor analysis, and internal consistency analysis. The tool was tested in a sample of 1024 healthcare professionals from diverse disciplines and levels of seniority working in Belgian hospitals.

**Results:**

The CLeaFA tool consists of 35 items across four factors: (a) departmental culture, (b) leadership style of the formal leader, (c) professional competence, and (d) team dynamics.

**Conclusions:**

The CLeaFA is a valid and reliable self‐assessment tool intended for both self‐reflection and offering potential use to evaluate contextual factors that influence their clinical leadership development, while also offering potential for use in training, management, and research. Future studies should further examine its applicability across diverse hospitals.

**Implications for Nursing Management:**

The four CLeaFA subscales may help nurse managers identify organizational and leadership‐related factors influencing clinical leadership and support leadership development planning. Further research is needed before using the CLeaFA to evaluate change or intervention responsiveness.

**Summary**
• What is already known
◦ Clinical leadership is essential for quality of care and innovation in healthcare.
◦ Contextual factors shape the development of clinical leadership.
◦ No validated tool exists to systematically assess these contextual factors.
• What this paper adds
◦ Development and validation of the CLeaFA, a self‐assessment tool for analyzing contextual factors influencing clinical leadership across hospitals

## 1. Introduction

Leadership in healthcare plays a crucial role in ensuring high‐quality patient care, fostering continuous professional development, and maintaining a resilient workforce [1]. Leadership refers to the ability to influence and guide others toward shared goals within a professional context [2, 3]. In healthcare, leadership can be enacted in formal and informal ways. Formal leadership is linked to designated managerial positions with organizational authority, whereas informal leadership emerges from professional expertise, credibility, and day‐to‐day influence within clinical practice [4, 5].

In clinical practice, leadership often emerges informally, with healthcare professionals influencing care processes and colleagues without holding formal leadership positions, a phenomenon commonly referred to as informal clinical leadership [3, 6]. It refers to the capacity of frontline healthcare professionals to influence clinical practice, enhance team performance, and contribute to care quality through their expertise, communication, and ability to apply clinical judgment in complex care situations [3, 5–7]. Unlike formal leadership, informal clinical leadership is typically enacted without formal authority and arises from day‐to‐day clinical interactions. It involves role modeling, promoting evidence‐based practice, facilitating collaboration, and driving small‐scale innovation at the point of care [8, 9].

Clinical leadership is closely linked to established leadership theories, particularly transformational leadership, which emphasizes inspiring others, fostering professional development, and promoting shared goals within teams [4]. Although often associated with formal leadership roles, these behaviors are equally evident in informal leadership, where healthcare professionals influence colleagues and care practices through expertise, role modeling, and day‐to‐day interactions [10]. One of the earliest instruments developed to measure staff nurse clinical leadership is derived from Kouzes and Posner’s model of transformational leadership [10], illustrating that clinical leadership is not only a contextual phenomenon but also shaped by leadership processes that encourage influence, collaboration, and professional growth. At the same time, the development of clinical leadership depends on both individual learning and supportive contextual conditions [11, 12]. Strategies to foster clinical leadership are most successful when they combine learning by knowing, observing, and doing, alongside a responsive work environment that enables healthcare professionals to enact leadership in daily clinical practice [11].

Healthcare professionals who actively engage in clinical leadership foster a culture of continuous improvement of quality of care by inspiring and supporting their colleagues while promoting evidence‐based practices and driving innovation [3, 13, 14]. Clinical leadership is vital for quality of care and innovation, yet its enactment is highly contingent on contextual factors. Several contextual factors have been identified that influence the development of clinical leadership among healthcare professionals. These include the following: (1) working environment, (2) leadership style of formal leaders, (3) professional identity, and (4) team dynamics [12].

First, the work environment of healthcare professionals can either facilitate or block the development of clinical leadership [11, 12]. An empowering working environment has been consistently linked to clinical leadership development, as it creates conditions that encourage initiative, critical thinking, and professional autonomy [5]. A recent realist review indicates clear context–mechanism–outcome configurations, showing that a responsive work environment (context) fosters the onset of ownership, confidence, and motivation (mechanisms), which leads to the presence of clinical leadership (outcomes) [11]. A workplace that fosters open feedback, offers continuous learning opportunities, and provides access to professional development has been shown to enhance professional identity and promote confidence and engagement in clinical leadership [15].

Second, the leadership style of formal leaders plays a decisive role in supporting informal clinical leadership [16]. Transformational leadership approaches, which emphasize mentorship, coaching, and participative decision‐making, have been shown to positively impact individual clinical leadership development among healthcare professionals [4]. Formal leaders who provide accessibility, encouragement, and strategic vision create environments where informal leaders can emerge and thrive [16, 17]. However, rigid management structures have been identified as significant barriers, limiting healthcare professionals’ autonomy and discouraging individual and informal clinical leadership initiatives [18].

Third, a well‐developed professional identity is also an essential factor in clinical leadership development. Research suggests that a strong sense of professional identity, shaped through education, mentorship, and professional recognition, fosters leadership behaviors and enhances role confidence [3, 15]. Conversely, a weak or underdeveloped professional identity may hinder clinical leadership development by reducing self‐confidence and willingness to take initiative [4, 12].

Finally, team dynamics are a decisive factor in the development of clinical leadership [12]. Teams characterized by collaboration, mutual trust, and shared decision‐making create environments where leadership can be distributed and exercised at multiple levels [19, 20]. Strong team cohesion has been associated with higher levels of informal clinical leadership engagement, whereas fragmented teams, unclear role expectations, and frequent staff turnover have been identified as barriers to clinical leadership development [19, 21].

Although contextual factors appear to play a crucial role in creating the conditions for clinical leadership, they do not fully explain why some healthcare professionals demonstrate stronger leadership behaviors than others. Personal characteristics such as clinical expertise, professional identity, self‐efficacy, educational background, leadership training, and years of professional experience have all been associated with the emergence and development of clinical leadership behaviors among healthcare professionals [5, 6, 11, 22].

Previous instruments, such as the Clinical Leadership Needs Analysis (CLeeNA) and the Clinical Leadership Analysis of Needs Questionnaire‐Barriers Scale (CLAN‐QBS), have focused primarily on identifying barriers to informal clinical leadership rather than on the conditions that actively enable it [23, 24]. A validated self‐assessment tool is needed to systematically identify the contextual factors that facilitate clinical leadership, thereby enabling organizations to design targeted interventions and create supportive environments for clinical leadership development. Previous qualitative research identified contextual factors influencing clinical leadership. Building on these findings, the present study operationalized these contextual actors into the Clinical Leadership Contextual Factors (CLeaFA) Self‐Assessment Tool and evaluated its psychometric properties.

### 1.1. Aim

This study aimed to develop and validate a self‐assessment tool that identifies contextual factors for the development of clinical leadership among healthcare professionals.

### 1.2. Ethics

Ethical approval was obtained from the ethics committees of the participating hospitals and covered both the survey and the Delphi component of the study (ONZ‐2023‐0526). For both components, informed consent was obtained at the start of the digital questionnaire. Participants, including the Delphi experts, were informed about the purpose of the study and were assured that participation was voluntary and that the collected data would be treated anonymously.

## 2. Methods and Results

The development and validation process comprised three phases: (1) item development, (2) scale development, and (3) psychometric scale evaluation, with a total of eight specific activities (Table 1). Details on item reduction and refinement are described in the main text.

**TABLE 1:** Phases and steps in the development of a clinical leadership influencing factors analysis (CLeaFA).

Phase	Period	Activities
1. Item development	April–Sept. 2023	1.1 Domain identification and item development • Initial item pool (*n* = 40 items) 1.2 Content validity (Delphi procedure) • Delphi Round 1: 40 items evaluated ⟶ 3 removed, 2 separated, 1 added (*n* = 40 items) • Delphi Round 2: 29 items evaluated; items optimized (*n* = 40 items) • Delphi Round 3: consensus on all items (*n* = 40 items)

2. Scale development	Oct. 2023–Jan. 2024	2.1 Survey administration (*n* = 40 items) 2.2 Item reduction and factor extraction (*n* = 35 items) • 4 factors identified: • Departmental culture (*n* = 8 items) • Leadership style of the formal leader (*n* = 10 items) • Professional competence (*n* = 6) • Team dynamics (*n* = 11 items)

3. Psychometric scale evaluation	Feb. 2024	3.1 Reliability analysis: • Cronbach’s alpha and McDonald’s *ω* • Leadership style: *α* = 0.90; *ω* = 0.92 • Team dynamics: *α* = 0.96; *ω* = 0.97 • Professional competence: *α* = 0.86; *ω* = 0.91 • Departmental culture: *α* = 0.94; *ω* = 0.96

### 2.1. Phase 1—Item Development

#### 2.1.1. Domain Identification and Item Development

##### 2.1.1.1. Methods

The aim of this phase was: (1) to conceptualize clinical leadership, (2) to identify contextual factors influencing the development of clinical leadership, and (3) to develop an initial set of items for the self‐assessment tool.

First, a literature search was conducted to conceptualize clinical leadership, serving as the conceptual foundation for the study and to identify existing instruments measuring clinical leadership and contextual factors influencing clinical leadership development. Second, the development of the item pool was strongly informed by a previously conducted qualitative study involving five focus group interviews with 41 healthcare professionals, including nurse assistants, licensed practical nurses, registered nurses, occupational therapists, recreational therapists, psychologists, and gerontologists in Flemish nursing homes [12]. This qualitative study explored which contextual factors healthcare professionals perceive as influencing the development of clinical leadership in practice. To ensure a diverse sample, all licensed nursing homes in East Flanders (*n* = 198) and West Flanders (*n* = 161) (Belgium) were invited to participate. Although these insights were generated in nursing homes and primarily reflect the perspectives of nursing and allied healthcare staff, previous research in hospitals and other hospitals has demonstrated similar contextual factors. This suggests that the findings are likely relevant across diverse healthcare environments and not unique to nursing homes [5, 16]. Finally, findings from both the literature and the qualitative study were translated into an initial set of items for the self‐report tool.

##### 2.1.1.2. Results

The literature search resulted in both the conceptual definition of clinical leadership and the identification of existing measurement instruments related to the context of clinical leadership development. Clinical leadership is defined as the ability to influence, inspire, and motivate others while actively engaging in patient care [3]. Clinical leaders serve as role models who exemplify professional values and norms, fostering improvements in care quality and team collaboration. Notably, their leadership is exercised without formal authority, highlighting their impact as frontline healthcare professionals [3, 22]. In addition, context is defined as the smallest unit of practice, referring to the immediate environment where healthcare professionals act and interact. Together, these definitions establish the basis for outlining the purpose of the domain within this study to identify the contextual factors that influence the development of clinical leadership among healthcare professionals. The literature search identified two validated instruments measuring contextual factors of clinical leadership development: the CLeeNA and the CLAN‐QBS. The CLeeNA assesses the specific needs of nurses in supporting the delivery of quality care, whereas the CLAN‐QBS focuses on identifying barriers to clinical leadership development [23]. Despite their contributions to understanding clinical leadership development, none of them systematically evaluate the contextual factors needed to develop clinical leadership among nurses.

The qualitative study of Nachtergaele et al. [12] identified four contextual factors of clinical leadership development: (1) work environment, (2) leadership style of the formal leader, (3) professional identity, and (4) team dynamics. An empowering work environment is essential, as it fosters professional growth through open feedback cultures, peer learning, and opportunities for leadership training. Such environments encourage continuous learning and reflection, making nurses more likely to develop and engage in clinical leadership. Second, the leadership style of formal leaders plays a crucial role in clinical leadership development. Leaders who are accessible, coaching‐oriented, and foster professional autonomy create a climate in which informal leadership can emerge. When formal leaders act as role models and actively mentor healthcare professionals, they provide a foundation for leadership development at all levels of the organization. Third, professional identity is also found to be a contextual factor. Healthcare professionals who receive structured leadership education, mentorship, and professional recognition are more likely to develop confidence in their leadership potential. When professionals understand their professional strengths and feel valued within their roles, they are more inclined to take initiative and contribute to decision‐making processes. Finally, strong team dynamics—characterized by collaboration, trust, and shared commitment—enhance leadership development by creating an environment where leadership can be exercised at multiple levels. When team members engage in mutual support, shared decision‐making, and constructive dialog, they foster a culture in which leadership is distributed rather than confined to hierarchical structures.

The domain identification phase resulted in the identification of four contextual factors, which formed the foundation for the item development phase, leading to the generation of an initial pool of 40 items.

#### 2.1.2. Content Validity

##### 2.1.2.1. Methods

To achieve expert input on the relevance and clarity of the items (Objective 1) and their alignment with the contextual factors of clinical leadership development (Objective 2), a three‐round Delphi procedure was conducted. In total, 25 experts from various healthcare and educational settings in Flanders, all with demonstrated expertise in clinical leadership, were invited every round, with 10 experts participating in the first round, 11 in the second round, and six experts in the third round (Table 2). Expert consultation took place between April and September 2023. Due to the anonymous nature of the survey, individual participation across rounds could not be tracked. After each round, group‐level feedback summarizing the results was provided to the invited experts via email before the subsequent round was initiated. The content validity index (CVI) was calculated to quantify the degree of agreement. Following Lynn’s method (1986), the cutoff value for acceptable agreement depends on the number of participating experts in each round. Accordingly, items with a CVI score below 0.78 in the first and second rounds (with 10 and 11 experts, respectively) or below 0.83 in the third round (with 6 experts) were revised or removed. Feedback provided by participants in the open‐ended fields was incorporated after each round to further refine the items.

**TABLE 2:** Composition of the Delphi expert panel by round.

	Round 1	Round 2	Round 3
Gender			
Male	4	5	2
Female	6	5	4
Age			
21–30 years	1	0	1
31–40 years	5	3	3
41–50 years	3	3	1
51–60 years	0	2	0
> 60 years	2	2	1
Healthcare experience			
0–5 years	1	0	1
6–10 years	2	2	3
11–15 years	2	1	0
16–20 years	1	1	0
21–25 years	2	3	1
> 25 years	1	3	1
Formal leadership position			
Yes	4	5	2
No	6	5	4
Function			
Nurse	2	2	2
Nurse managers	2	2	1
Nurse teacher	3	3	3
Advisory staff member	3	3	0
Scientific experience			
0–5 years	5	3	4
6–10 years	3	3	0
11–15 years	0	0	1
16–20 years	0	2	0
21–25 years	2	0	1
> 25 years	0	2	0

##### 2.1.2.2. Results

The first Delphi round involved 10 experts and initiated evaluation of 40 items. Based on the CVI scores and qualitative feedback, three items were removed. Additionally, two items were separated because they addressed overlapping themes and caused confusion when interpreted independently, thereby reducing the clarity of the instrument. One additional item was introduced to assess the professional proud of healthcare professionals. Consensus was reached on 11 items during this round.

The second Delphi round involved 11 experts and focused on further refining the remaining items based on their input. During this round, 29 items were revised according to expert feedback, and consensus was reached on 21 items, which were subsequently excluded from the third round of the validation process. Feedback from open‐ended responses was used to further refine the remaining items.

In the third and final round, six experts participated, reaching consensus on eight remaining items. This phase resulted in the first version of the CLeaFA measurement tool consisting of 40 items.

### 2.2. Phase 2—Scale Development

#### 2.2.1. Survey Administration and Sample Size

##### 2.2.1.1. Methods

The objective of this phase was to ensure the reliable and valid administration of the survey by minimizing measurement errors and selecting an appropriate sample size required for psychometric evaluation of the CLeaFA.

Data were collected between October and December 2023 from a convenience sample of healthcare professionals employed in 15 Belgian hospitals. All Belgian hospitals were invited to participate via email by the Belgian Federal Public Service for Health, of which 15 agreed to participate. Designated hospital contacts subsequently distributed the survey to eligible staff, aiming to ensure representation across professions, departments, and levels of seniority. Efforts were made to include participants of diverse ages, genders, and professional backgrounds. Determining an appropriate sample size for factor analysis is essential to ensure reliable and valid results. A commonly used guideline recommends at least 10 participants per item. Applied to the 40‐item CLeaFA, this implied a minimum sample size of 400 participants to achieve adequate statistical power [25].

The survey consisted of a demographic section, followed by the main survey, i.e., the newly constructed self‐reports on contextual factors. Items were rated on a six‐point Likert scale ranging from “*strongly disagree*” (0) to “*strongly agree*” (5). Example items were “In the department where I work, there is room to learn from mistakes”; “My formal leader adopts a coaching approach”; “In the team I work in, team members feel connected to each other”; and “The majority of my colleagues are competent in their job.”

##### 2.2.1.2. Results

The sample included a group of healthcare professionals, ensuring representativeness for psychometric evaluation (see Table 3).

**TABLE 3:** Demographic characteristics of participants within the target population.

Characteristics	Participants (*n*)	Percentage (%)
Sex		
Male	195	19.0
Female	828	80.9
X	1	0.1
Age		
< 25 years	47	4.6
25–34 years	296	28.9
35–44 years	286	27.9
45–54 years	256	25.0
> 55 years	139	13.6
Years of employment in the organization		
< 1 year	67	6.5
1–5 years	223	21.8
6–10 years	157	15.3
11–15 years	153	14.9
16–20 years	121	11.8
> 21 years	303	29.6
Years of employment in the department		
< 1 year	88	8.6
1–5 years	320	31.3
6–10 years	179	17.5
11–15 years	138	13.5
16–20 years	102	10.0
≥ 21 years	197	19.2
Highest degree		
Secondary degree	34	3.3
Undergraduate (EQF 5)	145	14.2
Bachelor (EQF 6)	594	58.0
Master (EQF 7)	181	17.7
PhD (EQF 8)	70	6.8
Employment percentage		
Part‐time (50%–99%)	502	49.0
Full‐Time (100%)	522	51.0

#### 2.2.2. Extraction of Factors and Item Reduction

##### 2.2.2.1. Methods

Statistical analyses were conducted using the statistical software R, Version 4.6.0 (2026‐04‐24 ucrt) [26]. First, negatively worded items were reverse‐coded to ensure consistent interpretation across all items. Prior to factor analysis, item distributions were visually inspected using histograms. Interitem correlations were calculated to evaluate the relationships between items and to identify potential redundancy or poorly performing items. Items with interitem correlations higher than 0.70 were critically evaluated by the research team for potential conceptual overlap [27]. Next, factor analyses were carried out using the package Lavaan [28, 29]. The analyses were conducted in three rounds of model refinement. At each round, the total sample (*N* = 1024) was randomly divided into two equal subsamples: an exploratory factor analysis (EFA) sample (*n* = 512) and a confirmatory factor analysis (CFA) sample (*n* = 512). EFA was conducted in the EFA sample to examine the underlying factor structure and guide item reduction. Given the ordinal response scale, models were estimated using diagonally weighted least squares (DWLS) with robust standard errors and scaled‐shifted test statistics [27, 28]. An oblique GEOMIN rotation was applied, allowing the factors to correlate. Factor solutions ranging from one to four factors were examined. Factor retention was based on a combination of comparative model fit (Comparative Fit Index [CFI], Tucker–Lewis Index [TLI], root mean square error of approximation [RMSEA], and standardized root mean square residual [SRMR]), factor loadings and communalities, factor interpretability, and theoretical coherence with the proposed conceptual framework. The CFI is considered excellent above 0.95 [30], as the TLI [31], RMSEA, and SRMR indicate an excellent fit between 0 and 0.05 [31], whereas values between 0.08 and 0.10 are considered to indicate a mediocre fit [30]. For factor loadings, cutoff values proposed. Cleare et al. (2018): 0.32 *poor*, 0.45 *fair*, 0.55 *good*, 0.63 *very good*, and 0.71 *excellent* [32]. Different factor solutions were compared to identify the most appropriate model fit. Model evaluation was based on factor interpretability, factor loadings, communalities, and overall fit indices. Items with factor loadings lower than 0.40 or with insufficient conceptual relevance were removed [27].

Following the EFA, CFA was performed in the CFA sample (*n* = 512) to evaluate the factor structure identified during the exploratory analysis. CFA models were estimated using DWLS with robust standard errors and scaled‐shifted test statistics. Model fit was evaluated using CFI, TLI, RMSEA, and SRMR. Robust corrections were applied to account for the ordinal and non‐normally distributed nature of the item responses.

The factor structure was refined iteratively. The original 40‐item model was first explored using EFA in the EFA sample and then evaluated using CFA in the CFA sample. Items with weak psychometric performance were reviewed, after which the reduced model was reexamined. This process continued until a conceptually interpretable and psychometrically supported factor solution was obtained.

##### 2.2.2.2. Results

Following the initial item screening based on interitem correlations, items with high interitem correlations (> 0.70) were critically evaluated by the research team for potential conceptual overlap. No items were removed at this stage, as the identified items were considered to represent conceptually distinct aspects of the construct.

In Round 1, an initial EFA was conducted on the 40‐item instrument in the EFA sample (*n* = 512). One‐ to five‐factor solutions were examined. Model fit improved progressively from the one‐ to the four‐factor solution, whereas extracting a fifth factor did not result in a meaningful further improvement in model fit. The four‐factor solution showed the best fit among the examined models, with a scaled CFI of 0.947, scaled TLI of 0.934, scaled RMSEA of 0.079, and SRMR of 0.040. In comparison, the three‐factor solution yielded a scaled CFI of 0.919, scaled TLI of 0.905, scaled RMSEA of 0.094, and SRMR of 0.053. The four‐factor solution explained 62.1% of the total variance.

The four‐factor solution was also the most consistent with the hypothesized multidimensional structure. Factor correlations ranged from 0.311 to 0.550, supporting the use of an oblique rotation.

Inspection of the individual items revealed that most items showed meaningful primary factor loadings. However, several items demonstrated weaker psychometric performance. Items 8 (manageable workload), 27 (top‐down leadership), 36 (staff turnover), and 39 (conflicting values and norms) showed a combination of low communalities, weak primary factor loadings, and/or substantial cross‐loadings.

The emerging four‐factor structure was subsequently evaluated using CFA in the CFA sample (*n* = 512). The model yielded a scaled CFI of 0.958, scaled TLI of 0.956, scaled RMSEA of 0.079, and SRMR of 0.062. The robust fit indices were less favorable (robust CFI = 0.828, robust TLI = 0.817, and robust RMSEA = 0.098), indicating that the overall model fit should be interpreted with caution. At the item level, most indicators showed moderate to strong standardized factor loadings. However, the four items already identified as problematic in the EFA again showed weak performance. Standardized loadings were −0.042 for Item 8, 0.432 for Item 27, −0.154 for Item 36, and −0.276 for Item 39. The corresponding residual variances were high, particularly for Items 8 (manageable workload), 36 (staff turnover), and 39 (conflicting values and norms), providing additional support for the concerns identified during the exploratory analysis.

In addition, Item 11 (rigid rules) showed comparatively weak performance, with a standardized CFA loading of 0.438 and a residual variance of 0.808. As this item had also shown a relatively low communality in the EFA, it was retained temporarily but flagged for further evaluation during the subsequent model refinement stage.

Taken together, the initial EFA and CFA supported further evaluation of a reduced 36‐item four‐factor model, with Items 8, 27, 36, and 39 removed and Item 11 retained for additional assessment (Tables 4 and 5).

**TABLE 4:** Comparison of EFA and CFA model fit statistics across three split‐sample rounds.

Round	Analysis	Factors	*χ* ^2^	df	Robust CFI	Robust TLI	Robust RMSEA	SRMR	Explained variance
1	EFA	4	2609.123	626	0.852	0.815	0.094	0.040	62.1%
1	CFA	4	3095.491	734	0.828	0.817	0.098	0.062	—
2	EFA	4	2410.355	492	0.870	0.834	0.101	0.035	69.0%
2	CFA	4	2630.369	588	0.850	0.839	0.097	0.059	—
3	EFA	4	2157.652	461	0.886	0.853	0.096	0.034	69.6%
3	CFA	4	2694.747	554	0.835	0.822	0.107	0.056	—

**TABLE 5:** Exploratory and confirmatory factor loadings of the retained items across three rounds.

Item	Verkorte term	EFA R1	CFA R1	EFA R2	CFA R2	EFA R3	CFA R3
*F1—Departmental culture*							
1	Feedback culture	0.497	0.682	0.746	0.683	0.683	0.676
2	Addressing conflicts	0.452	0.741	0.764	0.700	0.648	0.701
3	Growth stimulation	0.606	0.796	0.816	0.806	0.635	0.785
4	Peer learning	0.575	0.818	0.770	0.835	0.684	0.844
5	Exceeding basic care	0.635	0.746	0.867	0.771	0.731	0.722
6	Innovation climate	0.477	0.855	0.690	0.831	0.565	0.824
7	Learning from mistakes	0.422	0.858	0.612	0.866	0.517	0.840
8	Manageable workload	0.430	−0.042	—	—	—	—
9	Responsibility for decisions	0.590	0.742	0.729	0.726	0.571	0.737
10	Critically thinking	0.592	0.752	0.683	0.715	0.643	0.678
11	Strict rules	0.379	0.438	0.264	0.412	—	—

*F2—Leadership style of the formal leader*							
17	Peer role modeling	0.773	0.897	0.773	0.871	0.823	0.869
18	Participative decision‐making	0.800	0.905	0.766	0.898	0.838	0.884
19	Leader presence	0.754	0.827	0.757	0.771	0.754	0.803
20	Accessibility	0.862	0.908	0.818	0.902	0.834	0.894
21	Listening ear	0.868	0.927	0.847	0.908	0.852	0.917
22	Strength‐based leadership	0.777	0.941	0.769	0.927	0.777	0.922
23	Coaching leadership style	0.829	0.934	0.808	0.936	0.850	0.924
24	Departmental vision	0.732	0.870	0.690	0.850	0.709	0.845
25	Innovation support	0.732	0.925	0.761	0.901	0.739	0.908
26	Inspiring	0.689	0.891	0.673	0.867	0.652	0.880
27	Top‐down leadership	0.244	0.432	—	—	—	—

*F3—Professional competence*							
12	Colleague confidence	0.468	0.832	0.690	0.826	0.587	0.822
13	Professional competence	0.569	0.838	0.825	0.857	0.666	0.856
14	Job satisfaction	0.560	0.847	0.875	0.885	0.586	0.894
15	Organizational seniority	0.948	0.699	0.692	0.717	0.912	0.726
16	Continuous professional development	0.478	0.647	0.347	0.636	0.514	0.647
36	Staff turnover	−0.493	−0.154	—	—	—	—
37	Team seniority	0.894	0.729	0.787	0.712	0.873	0.741
39	Conflicting values and norms	−0.291	−0.276	—	—	—	—

*F4—Team dynamics*							
28	Role expectations	0.414	0.800	0.617	0.785	0.484	0.777
29	Team connection	0.996	0.902	0.875	0.918	0.953	0.899
30	Mutual trust	1.009	0.903	0.878	0.912	0.968	0.919
31	Shared goals	0.661	0.847	0.796	0.820	0.539	0.875
32	Team support	0.998	0.902	0.872	0.897	0.886	0.900
33	Role clarity	0.572	0.776	0.635	0.793	0.412	0.811
34	Peer role modeling	0.428	0.697	0.386	0.664	0.332	0.722
35	Team climate	0.831	0.902	0.723	0.861	0.781	0.887
38	Team expertise	0.614	0.841	0.871	0.848	0.613	0.856
40	Pride in profession	0.436	0.762	0.795	0.742	0.509	0.782

Following the removal of four items in the first round, the 36‐item instrument was subjected to a second round of factor analyses. For this analysis, the total sample (*N* = 1024) was randomly split again into two equal subsamples: an EFA sample (*n* = 512) and a CFA sample (*n* = 512).

EFA was conducted on the 36‐item instrument in the EFA sample. One‐ to four‐factor solutions were compared. Model fit progressively improved as additional factors were extracted. The four‐factor solution provided the best fit among the examined models, with a scaled CFI of 0.954, scaled TLI of 0.941, scaled RMSEA of 0.087, and SRMR of 0.035. The four‐factor solution explained 69.0% of the total variance.

Inspection of the factor structure nevertheless indicated that the four factors were not equally well defined. In particular, the third factor did not contain a clear set of items with strong primary loadings, suggesting that the four‐factor structure was not yet optimally differentiated. Item‐level evaluation also identified item 11 (rigid rules) as a weak‐performing item. This item showed a primary factor loading of 0.264 and a communality of 0.184, indicating that only a limited proportion of its variance was explained by the extracted factors.

The 36‐item four‐factor model was subsequently evaluated using CFA in the CFA sample. The model yielded a scaled CFI of 0.953, scaled TLI of 0.950, scaled RMSEA of 0.082, and SRMR of 0.059. Robust fit indices were less favorable, with a robust CFI of 0.850, robust TLI of 0.839, and robust RMSEA of 0.097, indicating mixed evidence regarding overall model fit.

Most retained items demonstrated moderate to strong standardized factor loadings. Item 11 (rigid rules) again showed the weakest performance, with a standardized factor loading of 0.412 and a residual variance of 0.830. In contrast, the remaining items generally demonstrated substantially stronger loadings on their respective factors.

Taken together, the findings from the second EFA and CFA provided additional support for removing item 11. The resulting 35‐item instrument was subsequently subjected to a final round of EFA and CFA (Tables 4 and 5).

Following the removal of 1 item in the second round, the resulting 35‐item instrument was subjected to a third round of factor analyses. The total sample (*N* = 1024) was randomly split again into two equal subsamples: an EFA sample (*n* = 512) and a CFA sample (*n* = 512).

EFA was conducted on the 35‐item instrument in the EFA sample. One‐ to four‐factor solutions were compared, with model fit progressively improving as additional factors were extracted. The four‐factor solution provided the best fit among the examined solutions, with a scaled CFI of 0.961, scaled TLI of 0.949, scaled RMSEA of 0.085, and SRMR of 0.034. The corresponding robust fit indices were CFI = 0.886, TLI = 0.853, and RMSEA = 0.096. The four‐factor solution explained 69.6% of the total variance.

The four‐factor solution was also more clearly interpretable than in the previous round. All four factors were represented by items with meaningful primary factor loadings, and the variance explained was distributed across the four factors. Factor correlations ranged from 0.414 to 0.595.

Inspection of the individual items showed an overall interpretable loading pattern. Some items demonstrated secondary loadings, reflecting conceptual overlap between related factors, but no remaining item showed the same combination of a very weak primary loading and low communality observed for the items removed in the previous rounds.

The 35‐item four‐factor model was subsequently evaluated using CFA in the CFA sample. The model yielded a scaled CFI of 0.948, scaled TLI of 0.944, scaled RMSEA of 0.087, and SRMR of 0.056. Robust fit indices were less favorable, with a robust CFI of 0.835, robust TLI of 0.822, and robust RMSEA of 0.107. Thus, the different fit indices provided mixed evidence regarding overall model fit, with CFI/TLI approaching commonly applied criteria and SRMR indicating adequate fit, whereas RMSEA and the robust indices indicated remaining model misfit.

At the item level, all retained items demonstrated moderate to strong factor loadings in CFA, ranging from 0.647 to 0.924. No item therefore showed a loading below 0.40. Factor correlations ranged from 0.482 to 0.818, with the strongest associations observed between Factors 3 and 4 (*r* = 0.818) and between Factors 1 and 4 (*r* = 0.796) (Table 6).

**TABLE 6:** Item‐level and subscale statistics for the CLEAFA.

Items (short versions)	Mean (SD)	Unique variance	Communality	Standardized CFA loading	McDonald’s *ω*
*F1—Work environment*					
Feedback culture	3.44 (1.15)	0.403	0.597	0.676	
Growth stimulation	3.34 (1.24)	0.372	0.628	0.785	
Peer learning	3.95 (0.95)	0.302	0.698	0.844	
Exceeding basic care	3.75 (1.12)	0.349	0.651	0.722	
Innovation climate	3.33 (1.24)	0.337	0.663	0.824	
Learning from mistakes	3.68 (1.05)	0.325	0.675	0.840	
Responsibility for decisions	3.90 (0.96)	0.434	0.566	0.737	
Critically thinking	3.87 (1.09)	0.393	0.607	0.678	
8 items		0.302–0.434	0.566–0.698	0.676–0.844	0.919

*F2—Leadership style of the formal leader*					
Role modeling	2.99 (1.49)	0.180	0.820	0.869	
Participative decision‐making	3.12 (1.49)	0.164	0.836	0.884	
Leader presence	3.20 (1.56)	0.322	0.678	0.803	
Accessibility	3.75 (1.32)	0.140	0.860	0.894	
Listening ear	3.55 (1.44)	0.148	0.852	0.917	
Strength‐based leadership	3.26 (1.42)	0.127	0.873	0.922	
Coaching leadership style	2.98 (1.52)	0.140	0.860	0.924	
Departmental vision	3.11 (1.46)	0.242	0.758	0.845	
Innovation support	3.02 (1.53)	0.198	0.802	0.908	
Inspiring	3.34 (1.44)	0.266	0.734	0.880	
10 items		0.127–0.322	0.678–0.873	0.803–0.924	0.973

*F3—Professional competence*					
Colleague confidence	3.61 (1.02)	0.405	0.595	0.822	
Professional competence	3.91 (0.95)	0.304	0.696	0.856	
Job satisfaction	3.50 (1.13)	0.312	0.688	0.894	
Organizational seniority	3.52 (1.24)	0.357	0.643	0.726	
Continuous professional development	3.42 (1.31)	0.619	0.381	0.647	
Team seniority	3.53 (1.22)	0.344	0.656	0.741	
6 items		0.304–0.619	0.381–0.696	0.647–0.894	0.905

*F4—Team dynamics*					
Addressing conflicts	3.00 (1.20)	0.359	0.641	0.701	
Role expectations	3.66 (1.06)	0.402	0.598	0.777	
Team connection	3.41 (1.22)	0.109	0.891	0.899	
Mutual trust	3.41 (1.18)	0.105	0.895	0.919	
Shared goals	3.75 (1.12)	0.358	0.642	0.875	
Team support	3.61 (1.18)	0.172	0.828	0.900	
Role clarity	3.68 (1.07)	0.452	0.548	0.811	
Peer role modeling	3.39 (1.23)	0.575	0.425	0.722	
Team climate	3.60 (1.23)	0.246	0.754	0.887	
Team expertise	3.39 (1.18)	0.281	0.719	0.856	
Pride in profession	3.78 (1.08)	0.405	0.595	0.782	
11 items		0.105–0.575	0.425–0.895	0.701–0.919	0.961
Total: 35 items		0.105–0.619	0.381–0.895	0.647–0.924	—

### 2.3. Phase 3—Psychometric Evaluation

#### 2.3.1. Reliability

##### 2.3.1.1. Methods

Internal consistency was evaluated separately for each subscale of the final 35‐item instrument. As equal factor loadings across items could not be assumed, McDonald’s omega (*ω*) was used as the measure of internal consistency rather than Cronbach’s alpha [25]. McDonald’s omega coefficients were interpreted using commonly applied thresholds, with values of ≥ 0.70 *considered acceptable*, ≥ 0.80 *good*, and ≥ 0.90 *excellent* [27].

##### 2.3.1.2. Results

Internal consistency was excellent across all four subscales of the final 35‐item instrument. McDonald’s omega coefficients ranged from 0.905 to 0.973. Work environment (F1) demonstrated excellent internal consistency (*ω* = 0.919), as did *Leadership style of the formal leader* (F2; *ω* = 0.973), *Professional/team context* (F3; *ω* = 0.905), and *Team dynamics* (F4; *ω* = 0.961). Overall, these findings indicate excellent internal consistency across all four subscales (Table 6).

## 3. Discussion

To facilitate the development of clinical leadership in hospitals, this study developed the CLeaFA tool, a self‐report tool that measures key contextual factors influencing this development. The tool is innovative and makes it possible to obtain a picture of contextual factors that help or hinder an individual from acting as a clinical leader and thereby provides input for setting specific interventions.

The CLeaFA consists of 35 items clustered into four factors: (1) *Departmental culture*, (2) *Leadership style of the formal leader*, (3) *Professional competence*, and (4) *Team dynamics*. Its construct validity is supported by both EFA and CFA, which confirmed the four‐factor structure and demonstrated that the items adequately represent the underlying factors of the contextual factors [33].

Unlike existing tools, which primarily focus on individual leadership competencies [3] or barriers to leadership emergence [5], the CLeaFA shifts attention to the contextual factors that enable or hinder clinical leadership development. This perspective aligns with growing evidence that leadership development in healthcare is shaped not only by individual competencies but also by the work environment, leadership style of formal leaders, and team dynamics [16, 34].

Accordingly, the CLeaFA provides a structured way to assess key contextual factors identified in the literature. The CLeaFA operationalizes elements such as departmental culture, characterized by openness to feedback, learning opportunities, and professional autonomy. Work environments that support continuous learning and reflective practice contribute to leadership identity formation and increased self‐efficacy, whereas hierarchical and rigid structures may inhibit leadership development [18].

Leadership style was identified as a key contextual factor, including coaching behaviors, accessibility of formal leaders, and shared decision‐making, as well as team dynamics within healthcare teams. These findings are consistent with prior research, demonstrating that transformational and empowering leadership styles foster engagement in informal leadership behaviors among healthcare professionals [4, 17, 35].

In addition, professional competence also emerged as a distinct contextual factor influencing clinical leadership. This factor encompasses healthcare professionals’ competence and confidence, job satisfaction, professional experience, and engagement in continuous professional development [36, 37]. These findings align with previous research identifying clinical expertise, self‐confidence, experience, and lifelong learning as important foundations for clinical leadership [11, 12]. Opportunities to develop expertise and engage in continuous learning may therefore strengthen healthcare professionals’ capacity to demonstrate clinical leadership in practice.

Finally, team dynamics emerged as key enablers of clinical leadership. Factors such as mutual trust, psychological safety, and shared goals have been identified as essential conditions for informal leadership emergence [19, 20, 38]. Psychologically safe environments promote open communication, learning, and innovation and are reinforced by trust, role clarity, empowering leadership, and targeted training [39]. High‐functioning interprofessional teams further strengthen leadership capacity by supporting collaboration, shared responsibility, and effective decision‐making [40–42].

Together, these findings highlight that clinical leadership development requires more than individual competence building; it depends on creating supportive and empowering environments. The CLeaFA tool enables organizations to identify strengths and areas for improvement in these contexts, providing a practical basis for targeted interventions. However, as a self‐assessment tool, it is important to acknowledge that responses may be influenced by subjectivity and social desirability despite offering valuable insight into healthcare professionals’ perceptions [27]. This study provides strong evidence supporting the psychometric properties of the CLeaFA, demonstrating high internal consistency [33] and a stable multidimensional structure comprising departmental culture, leadership style, professional competence, and team dynamics. Both EFA and CFA supported this structure, and all items met commonly accepted threshold (> 0.40), indicating their adequate contribution to the underlying four constructs [27, 32].

In terms of applicability, the CLeaFA offers a practical tool for healthcare organizations to assess contextual strengths and areas for improvement that influence clinical leadership development. This study extends previous qualitative research by translating previously identified contextual factors into a structured measurement instrument and evaluating its psychometric properties. It can support managers and educators in designing targeted clinical leadership development initiatives, facilitate reflective dialog within teams, and evaluate the impact of organizational interventions over time [11, 12]. A limitation of this study is that contextual factors were assessed through self‐assessment and therefore reflect healthcare professionals’ perceptions rather than objectively measured characteristics of the departmental culture, leadership style of the formal leader, and team dynamics. Nevertheless, these perceptions are relevant, as they directly influence how healthcare professionals experience support for clinical leadership development.

Despite these strengths, several limitations should be acknowledged. As participants were recruited exclusively from hospital settings, the generalizability of the findings to other healthcare contexts, such as primary care, mental health services, or long‐term care facilities, may be limited. Further research is therefore needed to examine the applicability and psychometric properties of the instrument across different healthcare settings. The current study focused on the factor structure and internal consistency of the CLeaFA. Other relevant psychometric properties, including test–retest reliability, convergent and discriminant validity, responsiveness to change, and measurement invariance, were not assessed. Future research should examine these properties to further establish the reliability and validity of the CLeaFA and to determine its applicability across different populations and healthcare settings. Longitudinal studies are also recommended to examine how targeted interventions influence organizational context, departmental culture, leadership style, and team dynamics over time, and to assess whether changes in CLeaFA scores reflect the tool’s sensitivity to clinical leadership development initiatives [27].

The CLeaFA offers nurse managers insight into contextual conditions that may facilitate or hinder the establishment of clinical leadership. Its four subscales can help structure reflection on the work environment, leadership, professional competence, and team dynamics, and guide priorities for leadership development.

The CLeaFA is primarily intended to be interpreted through its four separate subscale scores, as each subscale represents a distinct contextual factor influencing clinical leadership and provides specific insights relevant to both interpretation and practical application. By assessing departmental culture, leadership style of the formal leader, professional competence, and team dynamics, the self‐assessment tool is intended for both self‐reflection and offering potential use to evaluate contextual factors that influence their clinical leadership development, while also offering potential for use in training, management, and research.

## 4. Conclusions

The CLeaFA is a validated tool for self‐assessing contextual factors that influence clinical leadership development among healthcare professionals. It comprises 35 items organized into four factors: departmental culture (9 items), leadership style of the formal leader (8 items), and team dynamics (11 items), and demonstrates strong internal consistency.

By focusing on leadership‐enabling contextual factors, the CLeaFA offers a valuable contribution to the field. It provides a structured approach to identify strengths and areas for improvement within healthcare environments, thereby supporting targeted interventions in personal and professional development, practice, research, and training *t*. Ultimately, by enabling the systematic assessment of leadership‐enabling conditions, the CLeaFA serves as a key instrument for fostering clinical leadership capacity among healthcare professionals and organizations, thereby enhancing the quality of care.

**Nomenclature**
CLSAT: Clinical Leadership Self‐Awareness Tool
CONSORT: Consolidated Standards of Reporting Trials

## Author Contributions

The contributions of each author to this manuscript are detailed according to the CRediT (contributor role taxonomy): Sabrina Nachtergaele: conceptualization, methodology, validation, formal analysis, investigation, writing–original draft, visualization, and project administration. Lukas Billiau: software, data collection, methodology, formal analysis, and investigation. Nele De Roo: conceptualization, validation, formal analysis, investigation,and writing–original draft. Kristof Eeckloo: conceptualization, methodology, formal analysis, resources, and supervision. Annelies Bockstael: methodology, software, formal analysis, validation, data curation, and writing–review and editing. Mieke Embo: conceptualization, methodology, validation, writing–review and editing, and supervision. Veerle Duprez: conceptualization, methodology, validation, writing–review and editing, supervision, and resources.

## Funding

This research was partially supported by funding from the Belgian Federal Service for Health, Food Chain Safety, and Environment. The funding supported the distribution and analysis of the surveys.

## Conflicts of Interest

The authors declare no conflicts of interest.

## Data Availability

The data that support the findings of this study are available from the corresponding author upon request.
